# Glucagon-like peptide-1 receptor agonists in liver transplant recipients with diabetes: changes in glucose control and cardiometabolic risk factors

**DOI:** 10.3389/fendo.2025.1586941

**Published:** 2025-05-27

**Authors:** Valeria Grancini, Irene Cogliati, Gianfranco Alicandro, Andreina Oliverio, Clara Di Benedetto, Alessia Gaglio, Pietro Lampertico, Veronica Resi, Emanuela Orsi

**Affiliations:** ^1^ Fondazione Istituto di Ricerca e Cura a Carattere Scientifico (IRCCS) Ca’ Granda Ospedale Maggiore Policlinico, Endocrinology Unit, Milan, Italy; ^2^ Department of Pathophysiology and Transplantation, University of Milan, Milan, Italy; ^3^ Mother and Child Department, Pediatric Cystic Fibrosis Center, Fondazione Istituto di Ricerca e Cura a Carattere Scientifico (IRCCS) Ca’ Granda Ospedale Maggiore Policlinico, Milan, Italy; ^4^ Nutrition Research and Metabolomics Unit, Department of Experimental Oncology, Fondazione Istituto di Ricerca e Cura a Carattere Scientifico (IRCCS) Istituto Nazionale dei Tumori di Milano, Milan, Italy; ^5^ Division of Gastroenterology and Hepatology, Foundation Istituto di Ricerca e Cura a Carattere Scientifico (IRCCS) Ca’ Granda Ospedale Maggiore Policlinico, Milan, Italy; ^6^ CRC “A. M. and A. Migliavacca” Center for Liver Disease, Department of Pathophysiology and Transplantation, University of Milan, Milan, Italy

**Keywords:** glucagon-like peptide-1 receptor agonists, liver transplantation, diabetes mellitus, GLP1-RAs, post transplant diabetes mellitus

## Abstract

**Introduction:**

Data about efficacy and safety of GLP1 receptor agonists in liver-transplanted patients are lacking.

**Methods:**

Among a population of liver-transplanted individuals with diabetes, we evaluated 68 patients before, 6, 12 and 18 months after starting a GLP1RA-based therapy, as add on to metformin or insulin. We assessed glycemic control, body weight and composition (with bio-impedance analysis), liver fibrosis and steatosis (with transient elastography). Amylase, lipase levels and concomitant therapies were recorded at basal and follow up evaluations. Patients had an e-mail contact to report any adverse events.

**Results:**

We observed a significant decrease in fasting plasma glucose, HbA1c, weight, BMI, waist circumference. We demonstrated a reduction in total and LDL cholesterol. Liver stiffness decreased during the first 6 months. The rate of adverse events was low and the symptoms reported didn’t require any medical measures: 26.9% reported mild nausea, only 3 patients (7.69%) discontinued the drug dose due to gastrointestinal intolerance. No pancreatitis episodes were detected, amylase and lipase levels didn’t increase (despite concomitant calcineurin inhibitors). No adjustments in immunosuppressant therapy were reported. Among the 45 patients requiring insulin when a GLP1RA therapy was added on, 20 (33.2%) and 31 (45.5%) could suspend insulin therapy at, respectively, 6 and 18 months.

**Discussion:**

In conclusion, GLP1RA-based therapy can be considered safe and effective in a short-term follow up in liver-transplanted patients. Further studies are needed to assess the effects of this drugs on long term complications, such as renal impairment, cardiovascular events and all-cause mortality.

## Introduction

Liver is the second most commonly transplanted organ, representing 23.3% of all transplant procedures worldwide (1). Metabolic syndrome (MS) and diabetes mellitus (DM) are common complications following liver transplantation, affecting approximately 40% and 30% of patients, respectively (2–3). In 2013 and 2024, an International Consensus Meeting proposed the term “Post Transplant Diabetes Mellitus” (PTDM) to refer to DM diagnosed after surgery, which also includes the former definition “New Onset Diabetes After Transplantation” (NODAT) (4).

Prompt identification and treatment of PTDM is crucial due to the 2-3-fold increased risk of rejection, infections, cardiovascular events and all-cause mortality in these patients (2–5).

To date, data on the glycemic targets for this population are limited. Therefore, it is recommended to individualize the therapeutic goals according to the current guidelines for type 2 DM treatment (6, 7).

Insulin is the most used treatment for PTDM in the immediate post-transplant period because it is not susceptible to drug-drug interactions (6) and because of its manageability. In this specific context, immunosuppressant therapy is characterized by high amounts of steroids, requiring an intensive glucose-lowering therapeutic scheme. As glucocorticoids are slowly tapered to a minimal amount in kidney transplant recipients or withdrawal in liver transplant recipients, alternative therapies, such as metformin, sodium-glucose cotransporter 2 inhibitors (SGLT2i), or glucagon-like peptide 1 receptor agonists – GLP1Ras) can be considered.

After the immediate post-transplant period, when considering long-term glucose lowering therapy, some critical issues must be taken into consideration. Firstly, cardiovascular events are a major cause of morbidity and mortality in liver transplanted individuals (8) due to the increased incidence of metabolic syndrome, driven by excessive weight gain after surgery. In this context, DM has been demonstrated to be an independent risk factor for CV complications (9–11). Additionally, one of the biggest challenges in PTDM management is the possible drug-drug interaction between anti-hyperglycemic agents and immunosuppressant therapy (12). Calcineurin (cyclosporine and tacrolimus) and mTOR inhibitors (everolimus and sirolimus) are the most involved drugs. They have similar pharmacokinetic properties, being both eliminated by the cytochrome P450 enzymes CYP3A4, CYP3A5 and the efflux pump P-glycoprotein or ATP- binding cassette subfamily B member 1 (ABCB1) (7). Consequently, any drugs that inhibits or induce one of these enzymes can increase or decrease exposure to the current immunosuppressant therapy. On the other hand, cyclosporine, which can inhibit CYP3A4 and ABCB1, can increase exposure to several anti-hyperglycaemic agents.

Finally, calcineurin inhibitors can cause or worsen renal dysfunction due to their nephrotoxic effect, limiting the use of several non-insulin agents for DM treatment (13).

For long-term DM management, insulin it is not indicated as first line therapy, due to its known negative effects on weight gain in people at risk for MS (14, 15) and its neutral impact on cardiovascular risk in those at increased risk for cardiovascular events (16).

Metformin its currently considered the first-line therapy in people with type 2 DM and without CV complications (17). It is not involved in drug-drug interactions (18) but its use is significantly affected by the presence of renal failure and is contraindicated when glomerular filtration rate is below 30 ml·min−1·1.73 m-2 (19). A retrospective study demonstrated that metformin is safe in kidney-transplanted patients (20).

The efficacy, safety and CV protection of GLP1RAs have been widely demonstrated in people with type 2 DM and obesity (21–24). Due to their pharmacokinetic characteristics, these drugs undergo proteolytic degradation and glomerular filtration, and thus they are not involved in drug-drug interactions (25). On the other hand, they may cause slowed gastric emptying, potentially influencing immunosuppressant drugs absorption.

A potential relationship between GLP1RAs and acute pancreatitis in subjects with diabetes was suggested, for the first time, in 2008 by the US Food and Drug Administration (FDA), referring to 30 case reports (26). Since then, several observational studies investigated on this issue without providing any univocal evidences, with the main part of studies disproving a potential interrelation (27–35).

Incidence of AP in liver transplanted individuals is 1.5-8% (36–38). Transplant-related biliary complications requiring ERCP, HBV infection and previous intra-abdominal surgery have been recognized as major risk factors (39). Again, the use of tacrolimus, cyclosporine and mycophenolate mofetil as immunosuppressant therapy has been demonstrated to be a potential risk factor for AP (40–42). However, no data are currently available on the potential increased risk of AP in liver-transplanted individuals with diabetes and treated with GLP1RAs.

Currently, only a few studies were performed to investigate the use of GLP1RAs in liver-transplanted patients.

Although they demonstrated that GLP1RAs safely and effectively reduce HbA1c levels in transplant recipients, they were limited by small sample size, short duration, or focused mainly on kidney-transplant recipients (43–46).

To evaluate the effects of GLP1RAs on glycemic control and cardiometabolic risk factors, and the occurrence of adverse events, we followed a population of liver-transplanted individuals with post-transplant diabetes mellitus who were treated for 18 months.

## Methods

### Study design

We conducted a prospective cohort study including all patients who underwent liver transplantation at Fondazione IRCCS Ca’ Granda - Ospedale Maggiore Policlinico and who were diagnosed with diabetes and followed at Endocrinology Unit between January 2021 and December 2024.

At screening, all patients aged > 18 years and with PTDM treated with insulin, metformin or diet/lifestyle intervention were offered a GLP1-RA based therapy if indicated, according to diabetes treatment guidelines (sc semaglutide, sc dulaglutide or oral semaglutide) as add on to their current therapy. Exclusion criteria were: concomitant GLP1RAs or DPPi based therapies and absolute/relative contraindications to GLP1RAs. Protocol visits were performed 6, 12 and 18 months after enrollment.

The study complies with the Declaration of Helsinki. The research protocol was approved by the 129 Ethics Committee of the IRCCS Ca’ Granda – Ospedale Maggiore Policlinico Foundation (Prot. n. 516) and has been registered on ClinicalTrials.gov (Identifier nr: NCT02038571). informed consent was provided by each participant.

**Figure 1:**
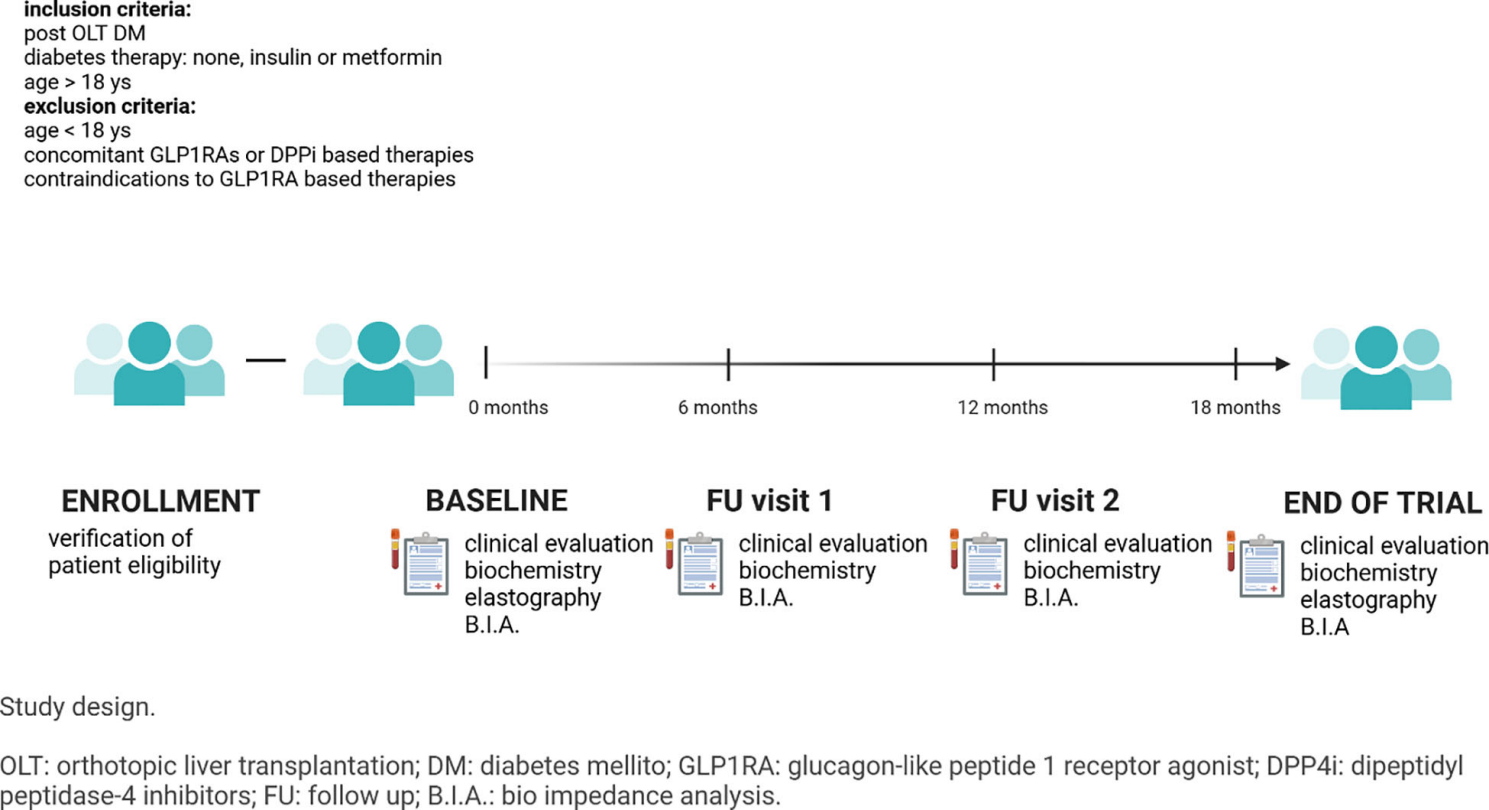
Study design.

Study flow chart is reported in **Figure 1**.

### Study procedures

At baseline, all participants underwent anthropometric evaluation (Waist circumference – WC, measured at the umbilicus level, and Body mass index – BMI, kg/m^2^, 47) and blood testing including fasting glycaemia (mg/dL), HbA1c (% and mmol/mol) Alanine transaminase (ALT, UI/L), aspartate transaminase (AST, UI/L), and gamma-glutamyl transferase (GGT, UI/L), alkaline phosphatase (ALP, UI/L), cholinesterase (CHE, UI/L), creatinine (Cr, mg/dl), total cholesterol (TC, mg/dl), high-density lipoprotein cholesterol (HDL, mg/dl), triglycerides (TG, mg/dl) were measured. Low-density lipoprotein cholesterol (LDL, mg/dl) values were calculated using the Friedewald formula (48). Glomerular filtration rate was calculated with the CKD-EPI formula (49). Body composition was assessed through a tetrapolar single frequency (50 kHz) bioelectrical impedance analyzer (Akern BIA 101 BIVA, Pontassieve, FI, Italy). Data obtained from bioimpedance were analyzed with the software BodyGram Plus. With this method we could estimate fat-free mass (FFM) and fat mass (FM).

Baseline evaluation was repeated at every follow up visit and at end-of-trial visit.

Baseline demographic and clinical characteristics, including sex, age, previous liver disease, concomitant illness and concomitant therapies were recorded.

Transient elastography (Echosens FibroScan Expert 630) was performed at baseline and at the end of the study to assess liver stiffness (50).

### Study outcomes

The primary outcomes of the study were changes from pre-treatment values in HbA1c and BMI. Secondary outcomes included changes in weight, WC, fasting glycemia, total cholesterol, LDL cholesterol, HDL cholesterol, triglyceride levels, and liver stiffness. Increases in amylase and lipase levels exceeding three times the upper limit of normal were considered indicative of a possible increased risk of pancreatitis associated with GLP1RA therapy.

### Statistical analysis

Baseline demographic and clinical characteristics. were summarized as frequency (percentages) for categorical variables and median ± standard deviation (SD) for continuous variables. Least square mean changes from baseline in study outcomes and corresponding 95% confidence intervals (CI) were estimated using linear mixed-effects regression models, which included a fixed effect for study visit and random intercepts and slopes. The statistical significance of the fixed effect was assessed using the likelihood ratio test, comparing the full model with a null model that included only the intercept and random effects. Statistical tests for the primary outcomes were conducted with a two-sided significance level of 0.025 to account for multiple comparisons. No multiple testing adjustment was applied to the statistical tests for the secondary outcomes. Analyses were done using the STATA 16 statistical package (StataCorp, College Station, TX, United States).

## Results

The study included 68 individuals with PTDM, whose main demographic and clinical characteristics are summarized in **Table 1**. The majority of study participants were male, with a mean age of 65 years, and 81% were overweight or obese. Only 59% had HbA1c values indicative of good glycemic control. Lipid profile abnormalities were common in this population, characterized mainly by low HDL cholesterol levels (38.2%) and elevated LDL cholesterol (33.8%) and triglycerides (32.3%).

**Table 1:** Patients’ characteristics at baseline.

Characteristic	N = 68
Male sex	61 (89.7%)
Age (years)	65.0 ± 6.9
Time from OLT (years)	6.8 ± 6.4
Body weight (kg)	83.8 ± 16.4
BMI (kg/m2)	29.1 ± 4.8
BMI category	
-Underweight	1 (1.5%)
-Normal weight	12 (17.7%)
-Overweight	29 (42.6%)
-Obesity	26 (38.2%)
Waist circumference (cm) ^a^	107.3 ± 12.5
≥102 cm in men and ≥88 in women	44 (64.7%)
Total cholesterol (mg/dL)	159.8 ± 30.5
≥200 mg/dL	6 (8.8%)
HDL cholesterol (mg/dL)	43.8 ± 10.0
<40 md/dL in men or <50 mg/dL in women	26 (38.2%)
LDL cholesterol (mg/dL)	87.8 ± 28.0
≥100 mg/dL	23 (33.8%)
Triglycerides (mg/dL)	144.3 ± 58.4
≥150 mg/dL	22 (32.3%)
AST (U/L)	22.8 ± 10.0
ALT (U/L)	27.9 ± 22.7
GGT (U/L)	51.4 ± 88.7
Creatinine (mg/dL)	1.2 ± 0.3
Albumin (g/DL) ^a^	4.3 ± 0.3
eGFR (mL/min per 1.73m^2^)	64.9 ± 19.1
Amylase (U/L) ^a^	73.1 ± 35.9
Lipase (U/L) ^a^	39.3 ± 16.5
Microalbuminuria^a^	13 (27.1%)
Fasting glycemia (mg/dL)	140.5 ± 38.7
HbA1c (%)^a^	6.8 ± 1.0
≥ 7%	27 (40.9%)
Liver stiffness (kpa) ^a^	6.8 ± 2.5
Fat Mass (%) ^a^	27.6 ± 7.5
Fat Free Mass (kg) ^a^	72.4 ± 7.4
Total Insulin	24.6 ± 27.5
DPP-4 therapy (% of yes)	15 (22.1%)
SGLT2 therapy (% of yes)	10 (14.7%)
GLP1 therapy (% of yes)	1 (1.5%)
ACE Inhibitor (% of yes)	20 (29.4%)
ARB (% of yes)	13 (19.1%)
CCB (% of yes)	20 (29.4%)
Beta-Blocker (% of yes)	34 (50.0%)
Diuretic (% of yes)	13 (19.1%)

BMI, Body Mass Index; HDL, High density lipoprotein; LDL, Low density lipoprotein; AST, Aspartate Aminotransferase; ALT, Alanine Aminotransferase; GGT, Gamma-Glutamyl Transferase; eGFR, estimated glomerular filtration rate; OLT, Orthotopic liver transplantation; ARB, Angiotensin II Receptor Blocker; CCB, Calcium Channel Blocker.

a Missing data for waist circumference (n=5), HbA1c (n=2), albumin (14), amylase (49), lipase (46), microalbuminuria (n=20), liver stiffness (n= 7), fat mass and fat free mass (21).

Among the patients in our population, all had completed the post-transplant steroid tapering, so none of them were on corticosteroid therapy at the time of evaluation.

62 patients started dulaglutide, 1 patient started injectable semaglutide 1 mg and 5 patients started oral semaglutide 7 mg. The choice of pharmacological treatment was guided by clinical considerations and patient preference. For the majority of patients, injectable dulaglutide was selected due to the ease of use of the delivery device and the absence of a need for titration to reach the therapeutic dose. However, 5 patients opted for oral semaglutide instead—despite the lack of available evidence at the time regarding its cardiovascular protective effects—as they refused subcutaneous therapy.

At baseline evaluation, one patient was treated with linagliptin and switched to dulaglutide 1.5 mg, one patient was treated with fixed combination liraglutide/degludec 15 U and, according to very low amount of GLP1RA assumed per day before basal evaluation, we considered the patient eligible for the study and switched him to dulaglutide 1.5 mg.

**Figure 2:**
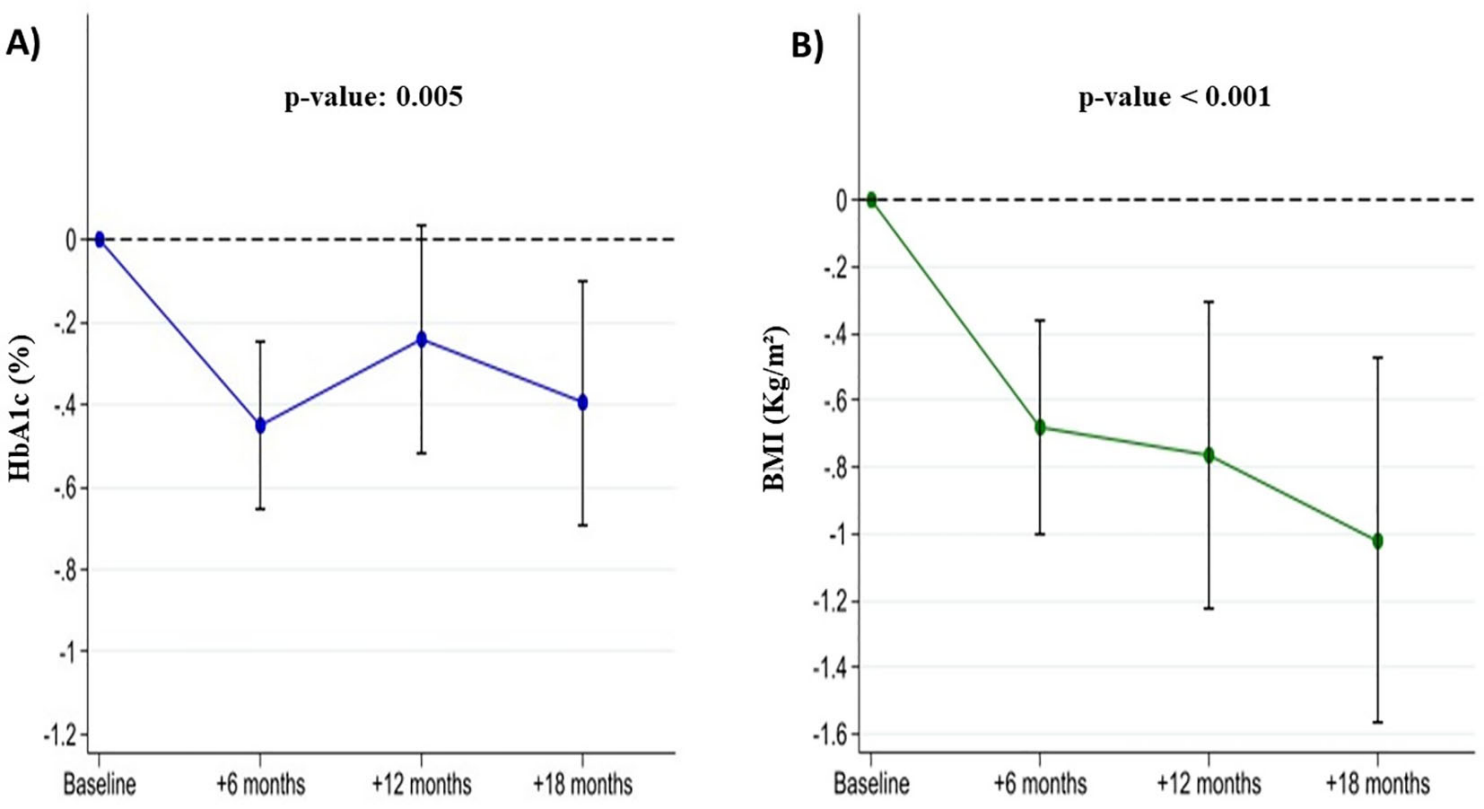
Changes in HbA1c (Panel **A**) and BMI (Panel **B**) in liver transplant recipients with post-transplant diabetes mellitus during GLP-1 receptor agonist treatment.

3 patients discontinued the treatment before follow up visit 1 due to gastrointestinal side effects. Among the 65 patients which completed the 18-months follow up, HbA1c decreased by an average of -0.5 percent point at the 6-month follow-up visit (95% CI: -0.6; -0.2) by -0.2 (95% CI: -0.5, 0.01) at the 12-month follow-up visit and by -0.4 (95% CI: -0.6; -0.2) at the 18-month follow up visit. BMI decreased by -0.7 kg/m^2^ (95% CI: -1.0; -0.3) at the 6-month follow-up and by -0.8 kg/m^2^ (95% CI: -1.1; -0.4) at the 12-month follow-up and -1.0 kg/m^2^ (95% CI: -1.3; -0.6) at the 18-month follow up visit (**Figure 2**).

**Figure 3:**
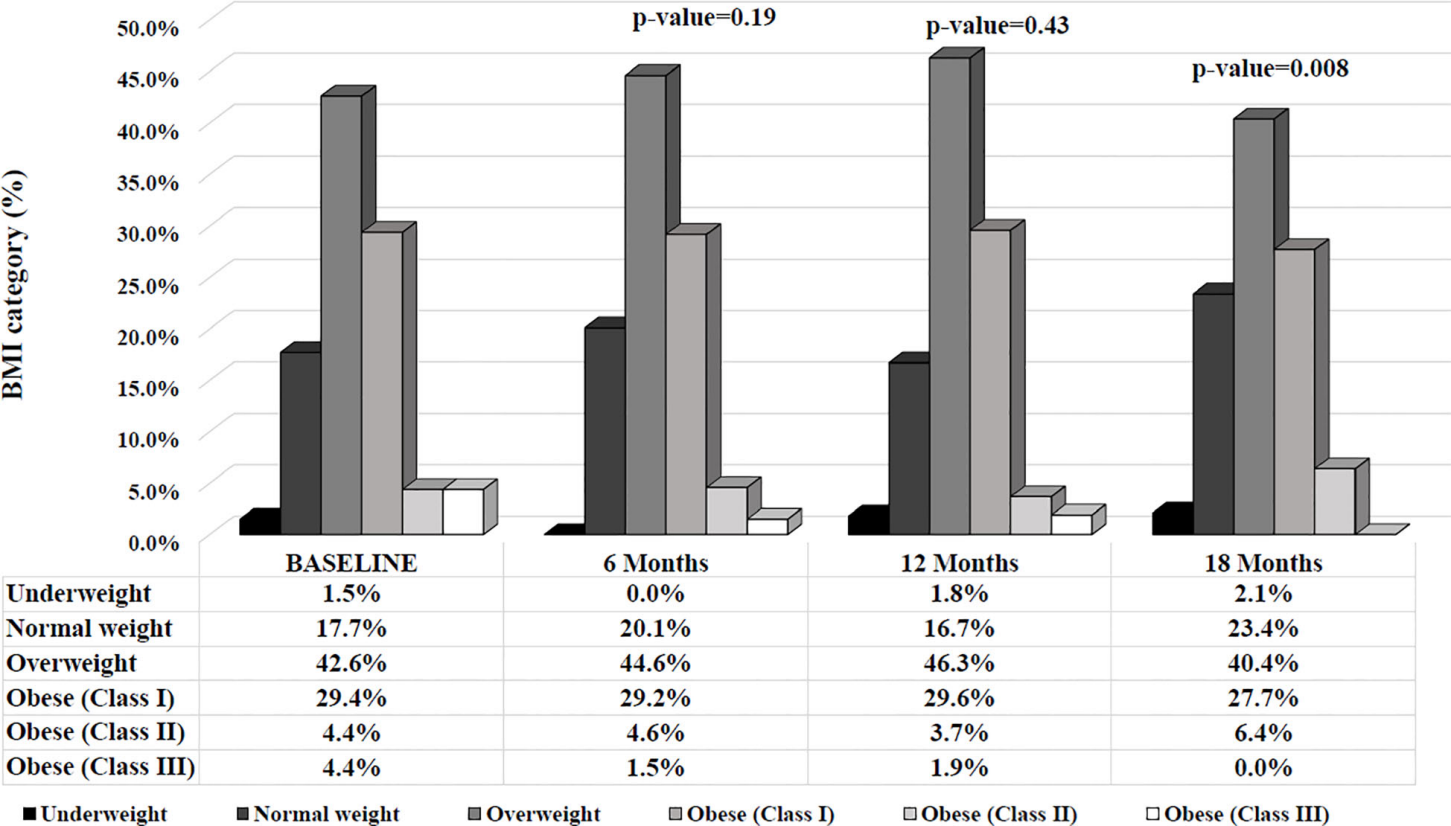
BMI category (percentage) along the 18 months of follow up visit.

Considering individuals according to their BMI categorization (under-, normal-, overweight and class I, II, III obesity), after 18 months, individuals were more likely to fall into a lower BMI category compared to baseline (**Figure 3**).

These reductions were accompanied by significant decreases in body weight (-1.9 kg at 6 months, p<0.001; -2.1 kg at 12 months, p<0.001; and -3.0 kg at 18 months, p<0.001), fasting glycemia (-19.8 mg/dL at 6 months, p<0.001; -14.0 mg/dL at 12 months, p=0.002; and -17.6 mg/dL at 18 months, p<0.001), and waist circumference (-2.1 cm at 6 months, p=0.003; -2.2 cm at 12 months, p=0.003; and -4.6 cm at 18 months, p<0.001). Significant reductions were observed in total cholesterol (p= 0.01) and LDL cholesterol levels (p=0.009). Total cholesterol decreased by -6.3 mg/dL at 6 months, p=0.04; by -5.8 mg/dL at 12 months, p=0.08; and by -11.2 mg/dL at 18 months, p=0.001. LDL cholesterol levels declined by -7.9 mg/dL at 6 months, p=0.01; by -7.2 mg/dL at 12 months, p=0.03; and by -10.7 mg/dL at 18 months, p=0.002. HDL cholesterol and decreased by around -0.6 mg/dL (p= 0.48) and -3.1 mg/dL (p=0.51), respectively.

In our study, 98% of transient elastographies reported an IQR/Med below 0.3 (mean 16 ± 2.3%). Liver stiffness decreased by an average of -0.6 kPa at 6 months (p=0.02) and by -0.5 kPa at 12 months (p=0.07); however, at the 18-month follow-up visit, liver stiffness increased slightly by an average of 0.1 kPa (p=0.8) (**Table 2**). Conversely, over the follow up period, CAP didn’t significantly change (+4.26 at 6 months, -3.11 at 12 months and +0.38 at 24 months).

**Table 2:** Mean changes in cardiometabolic risk factors and liver stiffness in liver transplant recipients with post-transplant diabetes mellitus during GLP-1 receptor agonist treatment.

Study outcome	Follow-up visit (months)	LSM (95% CI)	p-value
Weight (kg)	6	-1.9 (-2.8; -0.9)	<0.001
	12	-2.1 (-3.1; -1.0)	
	18	-3.0 (-4.1; 2.0)	
Waist circumference (cm)	6	-2.1 (-3.5; -0.8)	<0.001
	12	-2.2 (-3.7; -0.7)	
	18	-4.6 (-6.1; -3.1)	
Total cholesterol (mg/dL)	6	-6.3 (-12.5; -0.2)	0.01
	12	-5.8 (-12.3; 0.6)	
	18	-11.2 (-18.0; -4.4)	
LDL cholesterol (mg/dL)	6	-7.9 (-14.0; -1.9)	0.009
	12	-7.2 (-13.6; -0.8)	
	18	-10.7 (-17.4; -3.9)	
HDL cholesterol (mg/dL)	6	0.5 (-2.2; 3.3)	0.48
	12	-1.7 (-4.6; 1.2)	
	18	- 0.6 (-3.6; 2.5)	
Triglycerides (mg/dL)	6	-2.6 (-17.2; 11.9)	0.51
	12	7.9 (-7.4; 23.3)	
	18	-3.1 (-19.2; 12.9)	
Fasting glycemia (mg/dL)	6	-19.8 (- 27.9; - 11.7)	<0.001
	12	-14.0 (- 22.7; -5.3)	
	18	-17.6 (- 26.7; - 8.5)	
Liver stiffness (kpa)	6	-0.6 (-1.1; -1.0)	0.03
	12	-0.5 (-1.1; 0.1)	
	18	0.1 (-0.4; 0.6)	
Fat Mass (%)	6	0.5 (-1.4; 2.5)	0.81
	12	0.9 (-1.2; 3.1)	
	18	0.1 (-2.0, 2.2)	
Fat Free Mass (Kg)	6	-0.5 (-2.2; 1.1)	0.30
	12	-1.2 (-3.1; 0.6)	
	18	0.6 (-1.2; 2.4)	

CI, Confidence intervals; HDL, High density lipoprotein; LDL, Low density lipoprotein; LSM, Least squares means; OLT, Orthotopic liver transplantation.

Finally, we investigated any changes in the FAST score over time (50). We found a slide decrease in the two years follow up but we didn’t find any statistical significance (-0.01 at 6 months, -0.04 at 12 months and -0.03 at 24 months).

**Figure 4:**
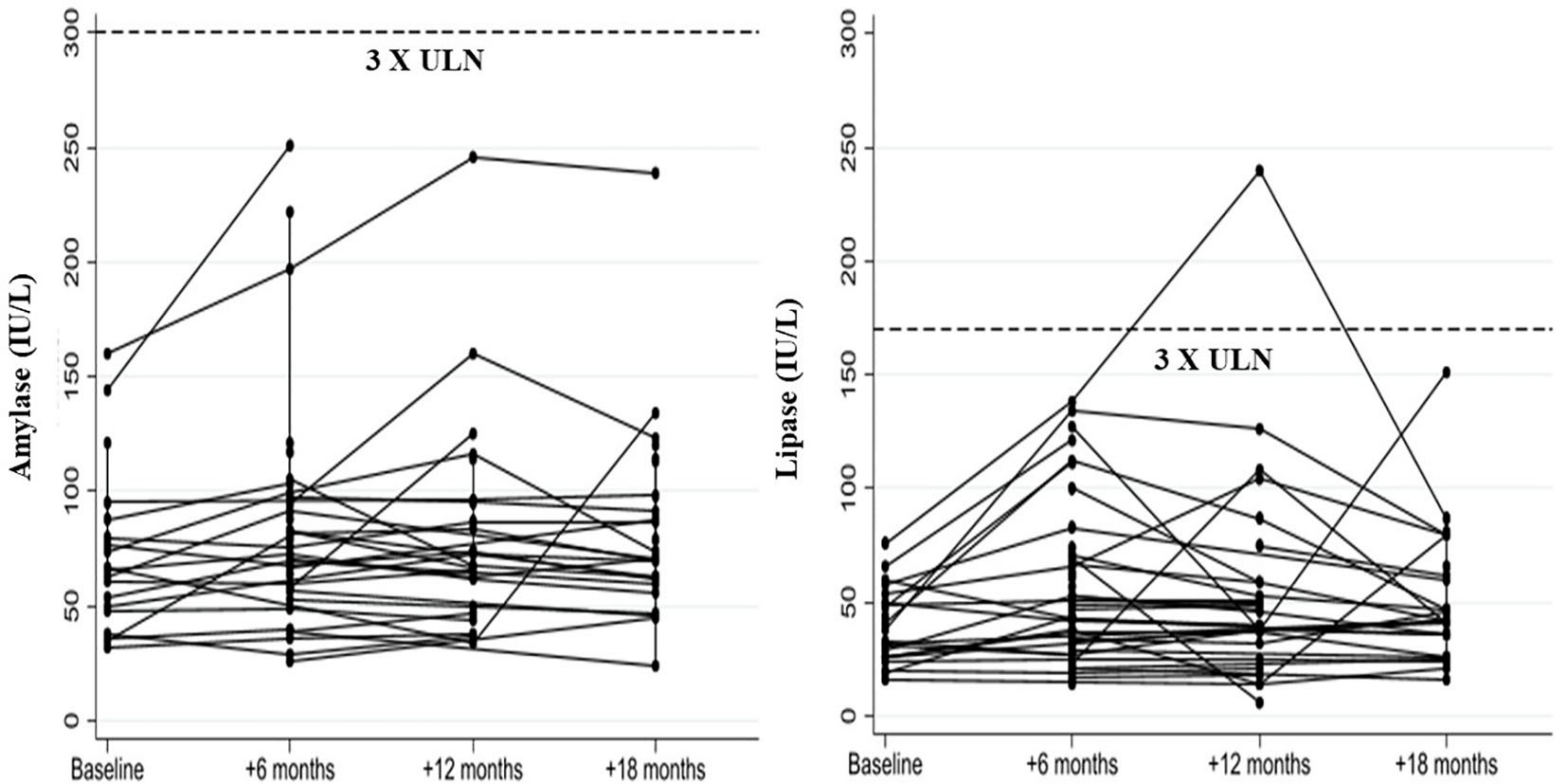
Changes in serum amylase and lipase levels in liver transplant recipients with post-transplant diabetes mellitus following GLP-1 receptor agonist treatment.

No study participant showed a serum amylase level > 3 times the ULN, while one patient developed a lipase level > 3 times ULN at the 12-month follow-up visit (**Figure 4**).

During the study, 2 patients had to modify their lipid-lowering therapy: 1 patient switched from simvastatin 10 mg to atorvastatin 10 mg, and 1 patient added ezetimibe 10 mg to the usual therapy with rosuvastatin 10 mg. In both cases, LDL cholesterol levels were not at target (according to their cardiovascular risk, LDL <70 mg/dl) at baseline assessment.

## Discussion

As discussed before, no specific guidelines are actually available on the treatment of PTDM.

Once steroid tapering is completed and long-term immunosuppressive therapy is established, a therapeutic strategy aimed at achieving the desired glycemic target and minimizing the risk of developing specific chronic PTDM complications must be planned. According to this, we set a HbA1c target of 7%.

The pharmacological treatment should therefore be tailored not only to achieve glycemic control but also to ensure cardiovascular protection and prevent the onset of overweight and obesity. In this context, GLP-1Ras could be considered first-line therapeutic agents.

On the other side, when treating people with PTDM, drug-drug interactions and potential specific side effects must be considered, such as delayed gastric emptying and the resulting interference with immunosuppressive therapy, the uncertain impact of these drugs on the increased risk of pancreatitis in patients already at risk due to the surgical procedure and concomitant tacrolimus therapy.

As previously mentioned, cardiovascular disease is one of the main causes of mortality and morbidity in liver transplant people (9). In our study, a 18-months follow up didn’t allow us to investigate the impact of GLP-1RAs on the incidence of cardiovascular events, but the data obtained from our population showed a clear positive impact on the well-known major risk factors, such as glycemic control, weight, and body composition.

Regarding concomitant therapies, all patients treated with SGLT2-i were already assuming the therapy at baseline. For this reason, we can hypothesize that the additional positive impact on glucose metabolism and body composition observed during the follow-up may be attributed to the addition of GLP-1RAs to the therapy.

Finally, we could demonstrate a positive effect of GLP-1RAs on hepatic fibrosis.

Regarding safety, in our population, no changes to the immunosuppressive therapy dosage were required, which aligns with previous studies by Singh et al. (44) and Thangavelu et al. (45). These findings, however, contrast with those reported by Liou et al. (51), where three kidney transplant individuals reduced their tacrolimus dosage upon initiating liraglutide therapy.

Among our patients, no major adverse events were reported. 26.9% of subjects experienced mild gastrointestinal effects, such as nausea, at the beginning of therapy, but only three patients required discontinuation of the treatment or dose reduction.

Lastly, an increase in amylase and lipase levels was observed after initiating GLP-1RAs, though this was not significant and did not require discontinuation of the medication.

Furthermore, the introduction of GLP1RAs enabled a reduction or suspension of concomitant insulin therapy in, respectively, 61 and 22% of individuals, thereby decreasing the risk of hypoglycemia and eliminating an additional factor negatively impacting body weight.

In conclusion, our study provides promising evidence on the safety and on the benefits of GLP-1RAs in glycemic control and cardiovascular risk factors control, in a population exclusively composed by liver transplanted individuals. Similar evidence has previously been reported only in studies based on small populations composed by organ transplanted people, where liver transplant recipients represented only a minority.

Further studies with larger populations and longer follow-up periods are needed to assess the impact of these medications on the incidence of cardiovascular events, the leading cause of mortality and morbidity in this population.

## Data Availability

The raw data supporting the conclusions of this article will be made available by the authors, without undue reservation.
